# Split Hollow Bulb Obturator to Rehabilitate Maxillary Defect: A Case Report

**DOI:** 10.7759/cureus.635

**Published:** 2016-06-09

**Authors:** Kasim Mohamed, Umamaheswari Mani, Prathibha Saravanakumar, S Prasanna Kumar, Ravikumar Arunachalam

**Affiliations:** 1 Prosthodontics, Faculty of Dental Sciences, Sri Ramachandra University, Chennai, India; 2 E.N.T., Sri Ramachandra University, Chennai, India

**Keywords:** hollow bulb, split design, sub-total maxillectomy, self retentive bulb, ball attachment

## Abstract

The rehabilitation of a maxillectomy patient involves meticulous treatment planning and designing. Lack of retention and facial support and limited mouth opening are the major issues that lead to functional and psychological trauma in post-maxillectomy patients. The successful rehabilitation of a maxillary defect includes restoring the function, esthetics, and a complete obturation of the defect, enabling the patient to feed without nasal regurgitation. This case report describes the fabrication of an obturator with a modified design, namely a split-antral hollow bulb obturator and oral part that is retained with a ball attachment, for a patient with right-side acquired maxillary defect due to recurrent myxoma. The primary advantage of this modification is enhanced facial support and a self-retentive antral obturation that improved the quality of life of the patient after an extensive maxillectomy.

## Introduction

Acquired maxillary defects are primarily due to neoplasm of the nasal and paranasal sinuses and osteomyelitis due to a bacterial or fungal infection. Odontogenic myxoma (OM) of the jaw most frequently occurs in the posterior mandible, but other locations such as the incisive sector, upper maxilla, and mandibular condyle must be considered [1]. The premolar to the ﬁrst molar region is the site of predilection in the maxilla, and the lesions usually occupy only one side, rarely crossing the midline [2]. OM is usually extensive, involving half of the maxilla or mandible including the ramus and the condyle. Surgical resection of such tumors results in impaired phonetics, mastication, swallowing, facial disfigurement, and a loss of communication between the nasal and oral cavity. Prosthetic and surgical rehabilitation offer functional and esthetic improvements to post-maxillectomy patients, and are essential to improving patients’ quality of life. However, the surgical reconstruction of maxillectomy defects is not always feasible due to potentially compromised general health in these patients.

Prosthetic rehabilitation improves a patient’s quality of life via an obturator bulb that seals the defect and a supporting prosthesis that replaces any lost teeth. For such patients, the fabrication of an obturator prosthesis offers the possibility of immediate and adequate dental rehabilitation [3]. The retention and stability of the obturator can be highly compromised due to the loss of teeth and bony structures, especially in total maxillectomy patients. Retention can be achieved by various aids such as anatomical undercuts, direct retainers, intracoronal attachments, extracoronal attachments, and implants. The attachment-retained prosthesis is preferred over direct retainers where esthetics is a concern, especially in the anterior region. A post-resection obturator denture with attachment improves retention and offers a level of resilience to compensate for stress from mastication as well as prevents food impaction into the defect [4].

Function and esthetics are achieved by use of a conventional 2-piece hollow bulb. In extensive defects like Type IIIA maxillectomies where the remaining structures are minimal to support facial structures, even the eyeball, prosthetic management is challenging. In such defects, the conventional obturator design will not meet the requirements of function and facial support. This article highlights and describes the importance of a modified obturator design consisting of a split self-retentive antral hollow bulb. The superior part of the bulb provides obturation and support to the eyeball, minimizing the facial disfigurement, and the inferior part acts as an extension of the oral part that aids in the retention of the superior bulb and mastication.

## Case presentation

**Figure 1:**
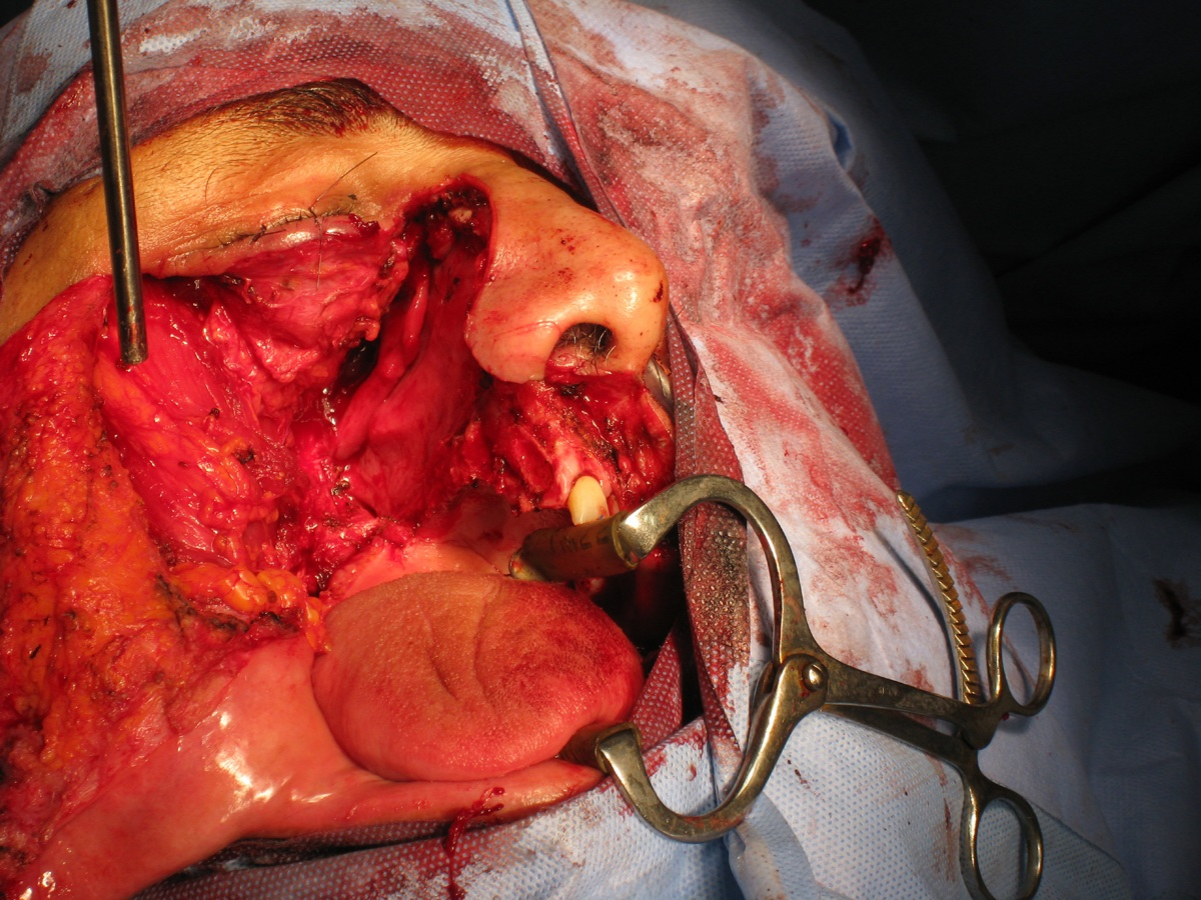
Surgical Excision of the Defect

A 48-year-old male was referred from the Ear, Nose, and Throat Department for fabrication of an immediate surgical obturator. Patient history revealed swelling of the right side of the face measuring approximately 7 cm × 6 cm, extending from the lower eyelid up to the upper lip. He had a right-sided nasal obstruction, a loss of sensation in the right maxillary region, and occasional blood-stained nasal discharge from the right nostril. Extraocular movement and vision were normal. Previous biopsy reports and fine needle aspiration cytology (FNAC) of the mass indicated a sarcoma. However, the previous slide was reviewed by the pathologist, who then suggested that the tumor could be a myxoma. A postoperative summary revealed that the tumor was excised via a Weber-Ferguson incision and an extended maxillectomy with wide excision of the tumor was performed, as shown in Figure 1.

**Figure 2:**
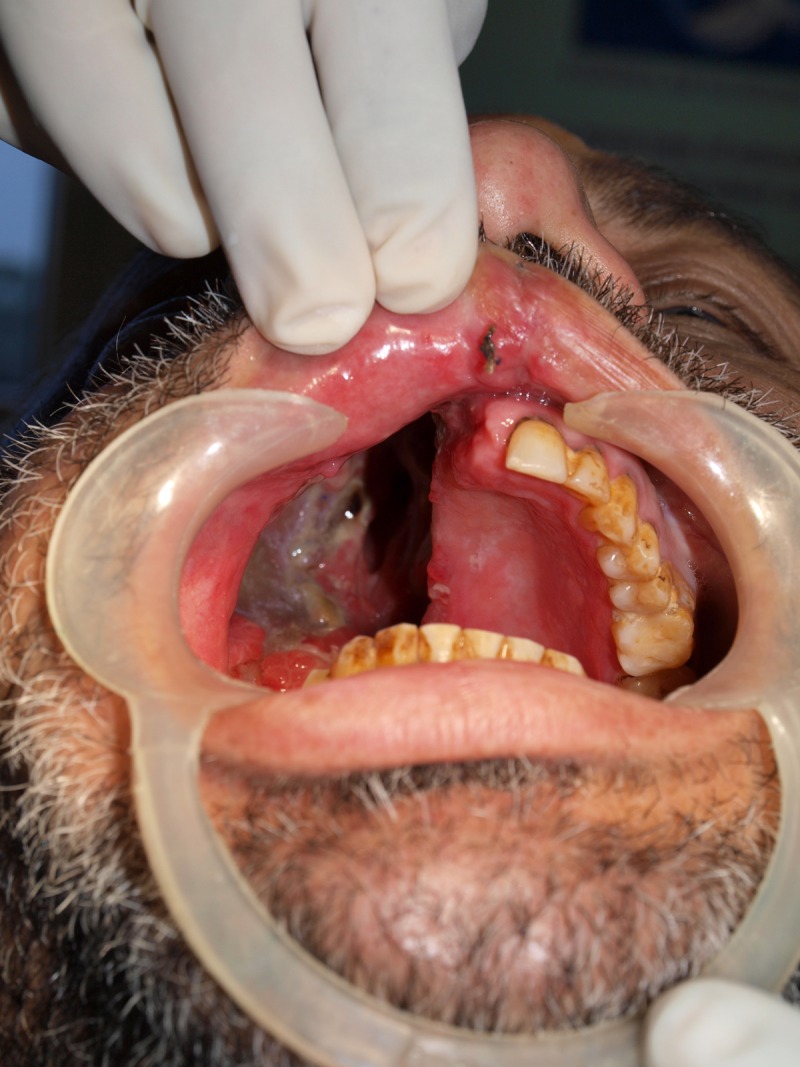
Intraoral Defect After Right Total Maxillectomy Intraoral view of the defect showing the extension of the defect and remaining hard tissue available for rehabilitation.

An analysis of a frozen tissue section was suggestive of myxoma. Although the tumor was extending into the orbit and infiltrating the periorbita, the eye was spared because of the benign nature of the tumor and the patient refused consent for enucleation. An immediate surgical obturator was inserted, and the patient was started on oral feeds. Two weeks following the operation, a delayed surgical obturator was fabricated to both support the flap and eyeball due to the extensive resection involving the orbital floor and enable the patient to feed without nasal regurgitation. Usually, a delayed surgical obturator is a single piece prosthesis. Due to the extensive nature of this intraoral defect (as shown in Figure 2), the obturator was fabricated in two parts, consisting of a hollow antral part and an oral part.

This modified surgical obturator served as the oral part of a delayed surgical obturator. The oral part of the delayed surgical obturator was modified into an interim obturator three weeks after the surgery that provided anterior esthetics. One month later, a wound defect approximately 1 cm below the right lower eyelid was noted, and the forehead flap cover was used to close the defect.

### Definitive obturator

Four months after the operation, a maxillary definitive obturator opposing the dentate mandibular arch was planned. The design consisted of a split antral bulb and an oral part. The hollow antral part was split into two pieces: a superior hollow bulb that occupied the superior two-thirds of the defect and an inferior hollow part that occupied the inferior one-third of the defect and was an extension of the oral part of the obturator. The oral part of the obturator included a splinted metal ceramic prosthesis with a ball attachment (Rhein 83, New York, USA) on the left maxillary central incisor. The cast partial framework included a complete palate design with embrasure clasps on the remaining posterior abutment teeth on the left side of the maxillary arch. Informed consent was obtained from the patient after the treatment sequence, and the limitations of maxillary obturators were explained to him.

Fabrication of Definitive Obturator

**Figure 3:**
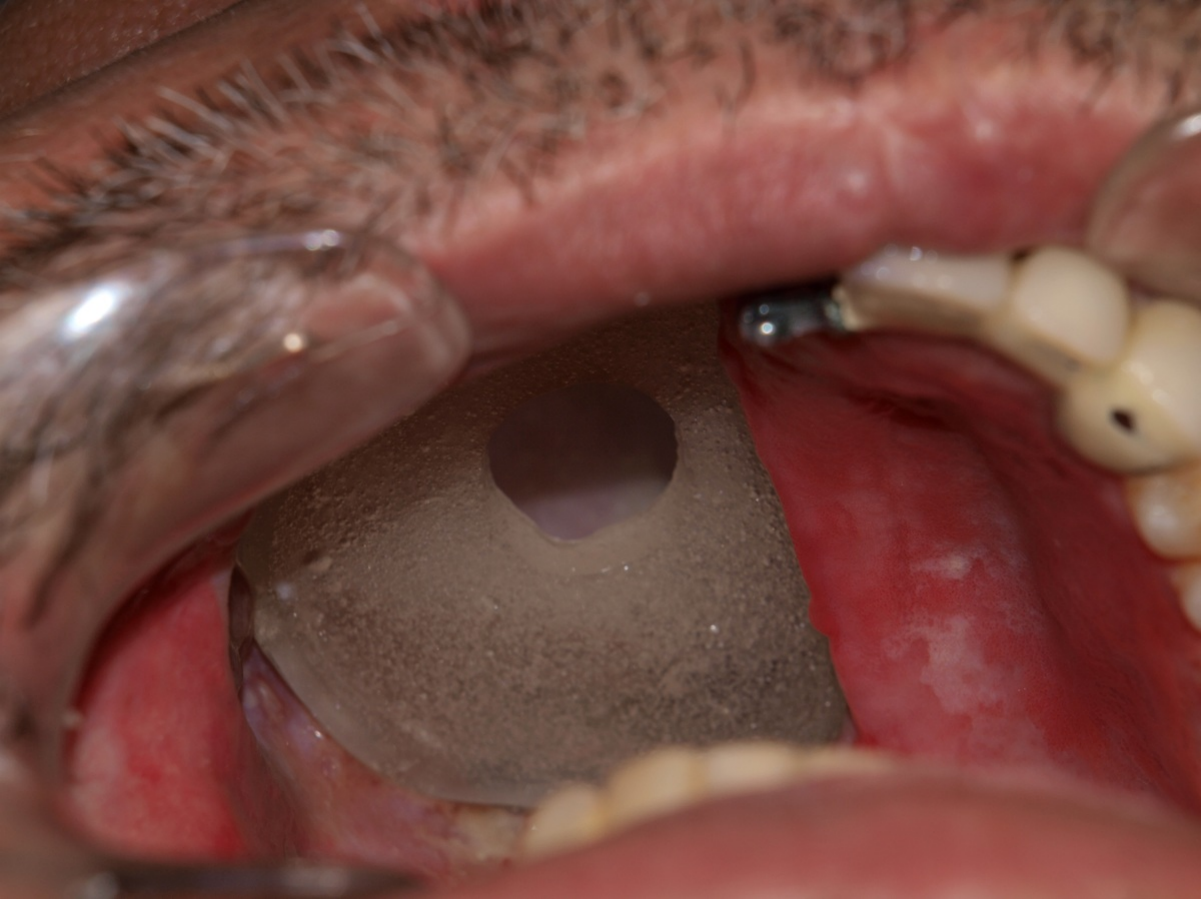
Superior Part of Split Antral Bulb with Perforation The figure shows the first part of the split design obturating the superior part of the defect. Note: The inferior part of the defect is still uncovered to be occupied by the second part of the bulb.

Antral Part: The antral part of the delayed surgical obturator was used as a tray to record the finer details after adequate healing of the defect area had occurred. The impression obtained was processed (heat-cure clear acrylic, DPI, Mumbai, India) to obtain a hollow bulb. The hollow bulb was finished and tried on the defect. It was modified to engage only the superior two-thirds of the defect. The lid (fabricated with a self-cure acrylic material, DPI, Mumbai, India) was perforated in the center to help the patient easily remove the bulb with his finger, as shown in Figure 3.

The superior part of the antral bulb was passive. It functions only when pushed by the second inferior part to engage the defect and support the eyeball. This inferior one-third of the antral bulb is an extension of the oral part. This split-antral bulb design is self-retentive within the defect.

**Figure 4:**
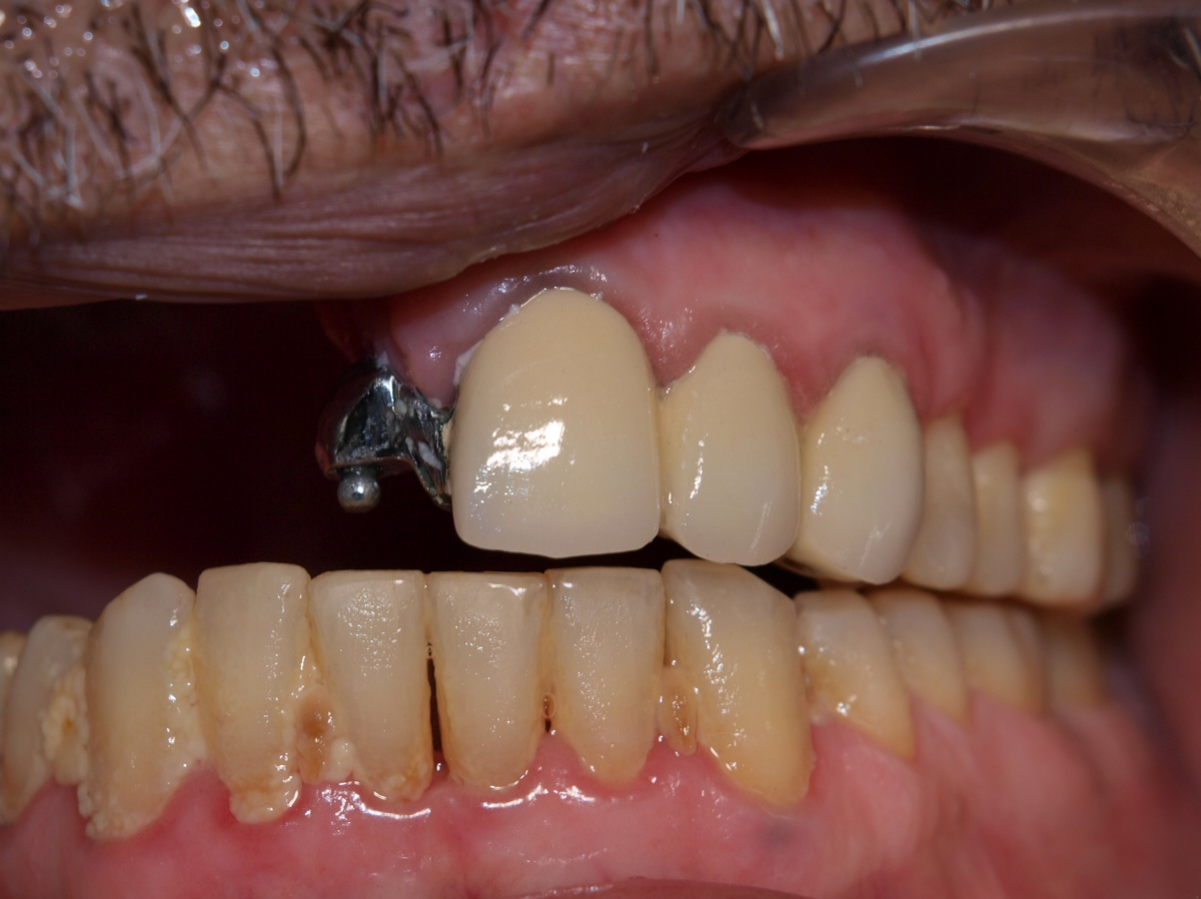
Splinted Metal Ceramic Prosthesis with a Ball Attachment The prosthesis is tried prior to impression-making of remaining defect and oral part.

Oral Part: The oral part comprised a cast partial maxillary removable denture replacing the teeth from the right central incisor to right maxillary second molar, and was retained with a ball attachment on the left central incisor and an acrylic part to engage one-third of the defect area. Tooth preparation was done on teeth 21, 22, and 23 to receive a ceramic metal prosthesis with a ball attachment. A final impression was recorded for the fabrication of the prosthesis. The final metal ceramic prosthesis with the male component was tested for fit, as shown in Figure 4.

**Figure 5:**
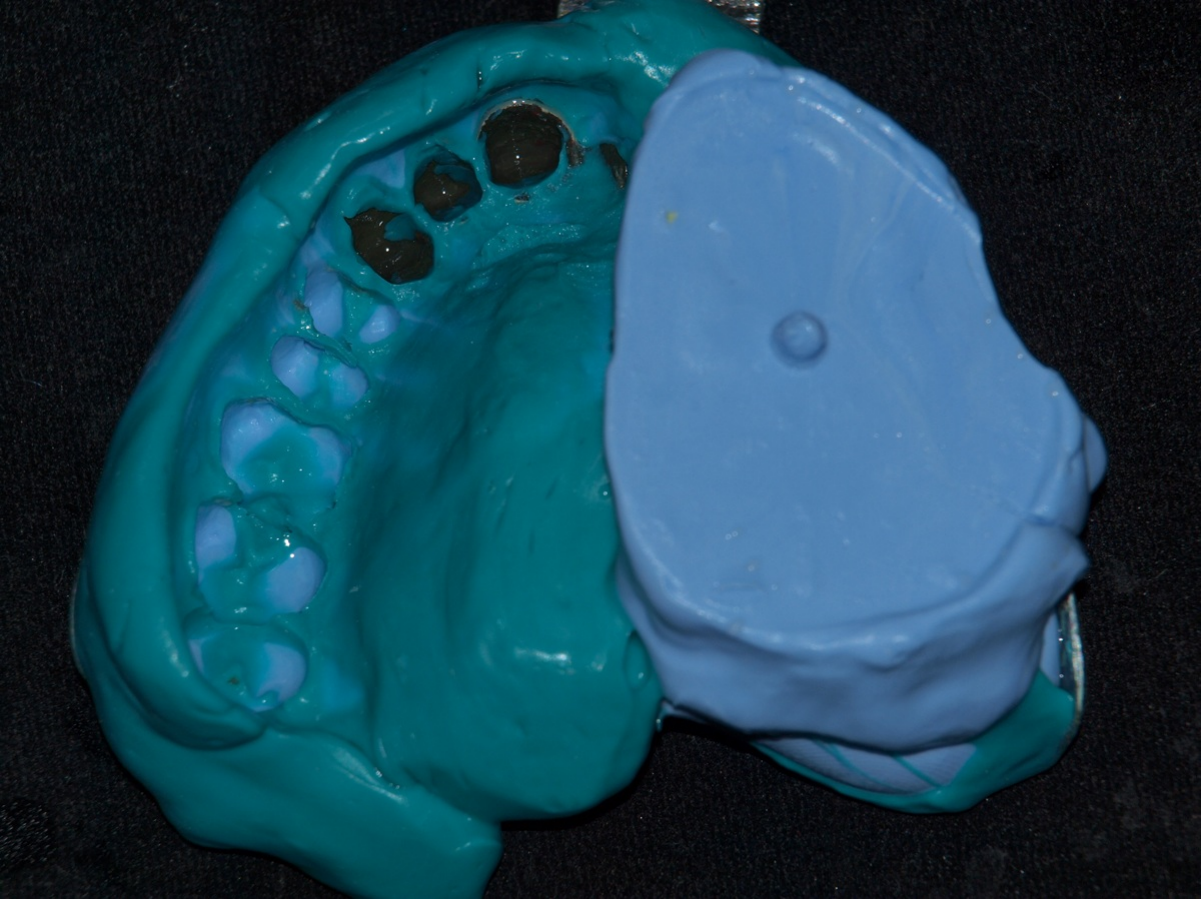
Record of Final Impression for Fabrication of the Oral Part and the Inferior Antral Bulb The impression of the inferior part of antrum was recorded in putty after masking the perforation on the superior bulb. The impression of the remaining teeth and prosthesis was recorded in light body or low viscosity.

The superior part of the antral bulb was inserted. The final impression was recorded with a polyvinyl siloxane (Aquasil, Dentsply, Germany), which included the inferior one-third of the defect, the remaining teeth, and the prosthesis with attachment, as shown in Figure 5.

**Figure 6:**
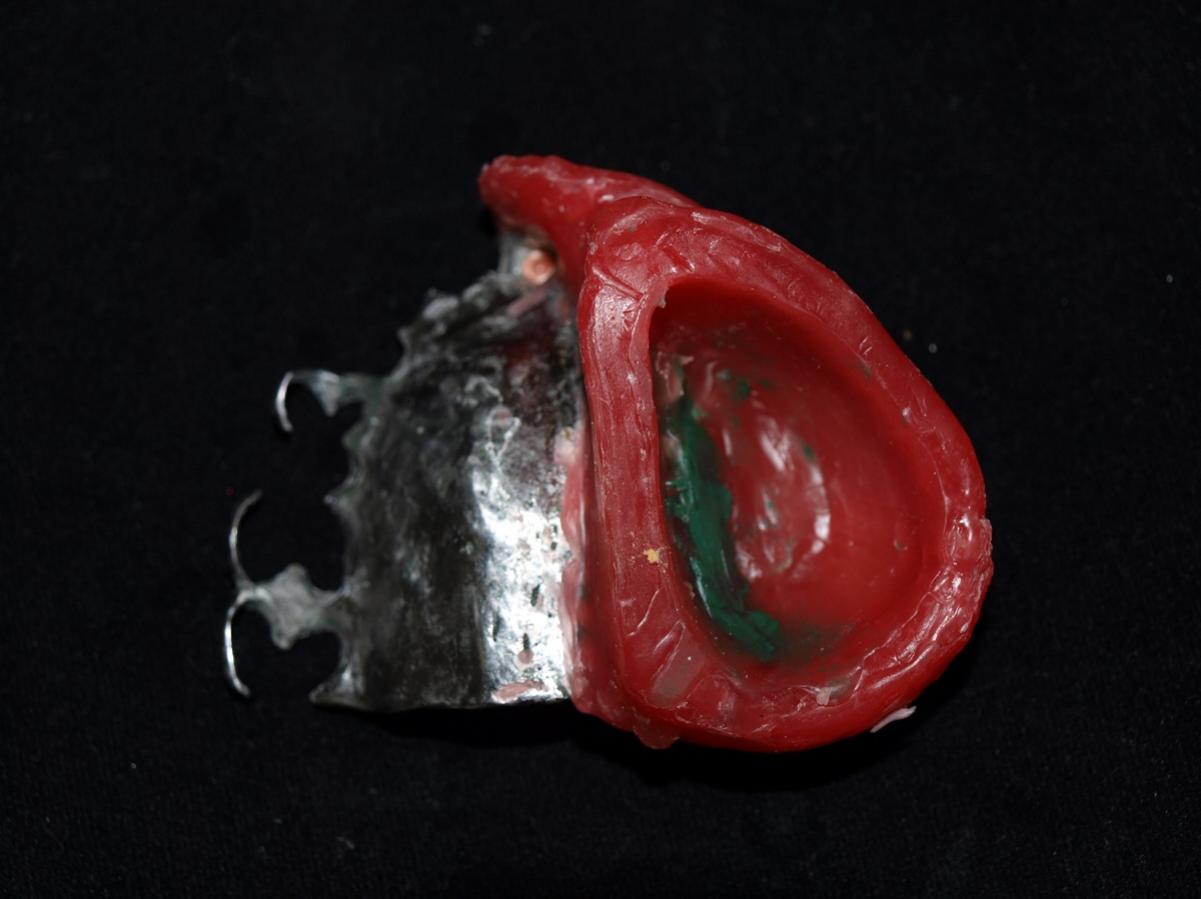
Trial Denture with the Intaglio Surface of the Oral Part and Inferior Bulb The inferior part of the split bulb is made hollow during the wax try-in appointment to evaluate the retention of prosthesis. The borders are contoured in modeling wax to accomodate or sit on the superior bulb.

The frame was fabricated and the jaw relationship was recorded on it. The fit of the attachment, occlusion, comfort, and esthetics were verified during the wax try-in appointment. The intaglio surface of the oral part on the defect side was hollowed further, which is shown in Figure 6, to reduce the weight of the final prosthesis and enhance the retention.

**Figure 7:**
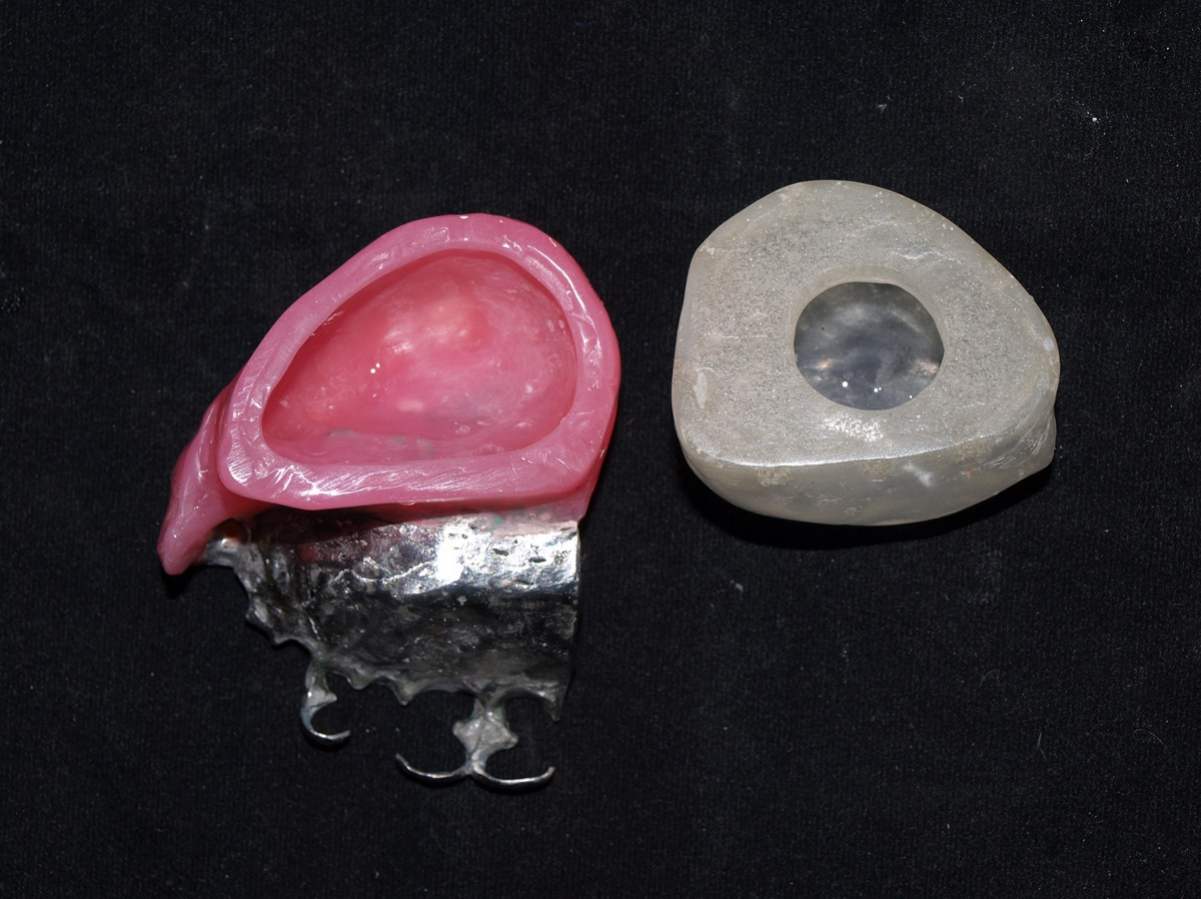
Finished and Polished Oral Part and Split Antral Bulb The picture shows the intaglio surface of the oral part and the superior part of the split bulb.

Only 3 mm to 4 mm of wax were spared to hold the teeth in their proper position. The framework with the wax pattern was acrylized (heat-cure acrylic, DPI, Mumbai, India). Both the split hollow bulb and the oral part were polished before final insertion, as shown in Figure 7.

During the insertion, the hollow antral part of the obturator was inserted first, followed by the oral part.

**Figure 8:**
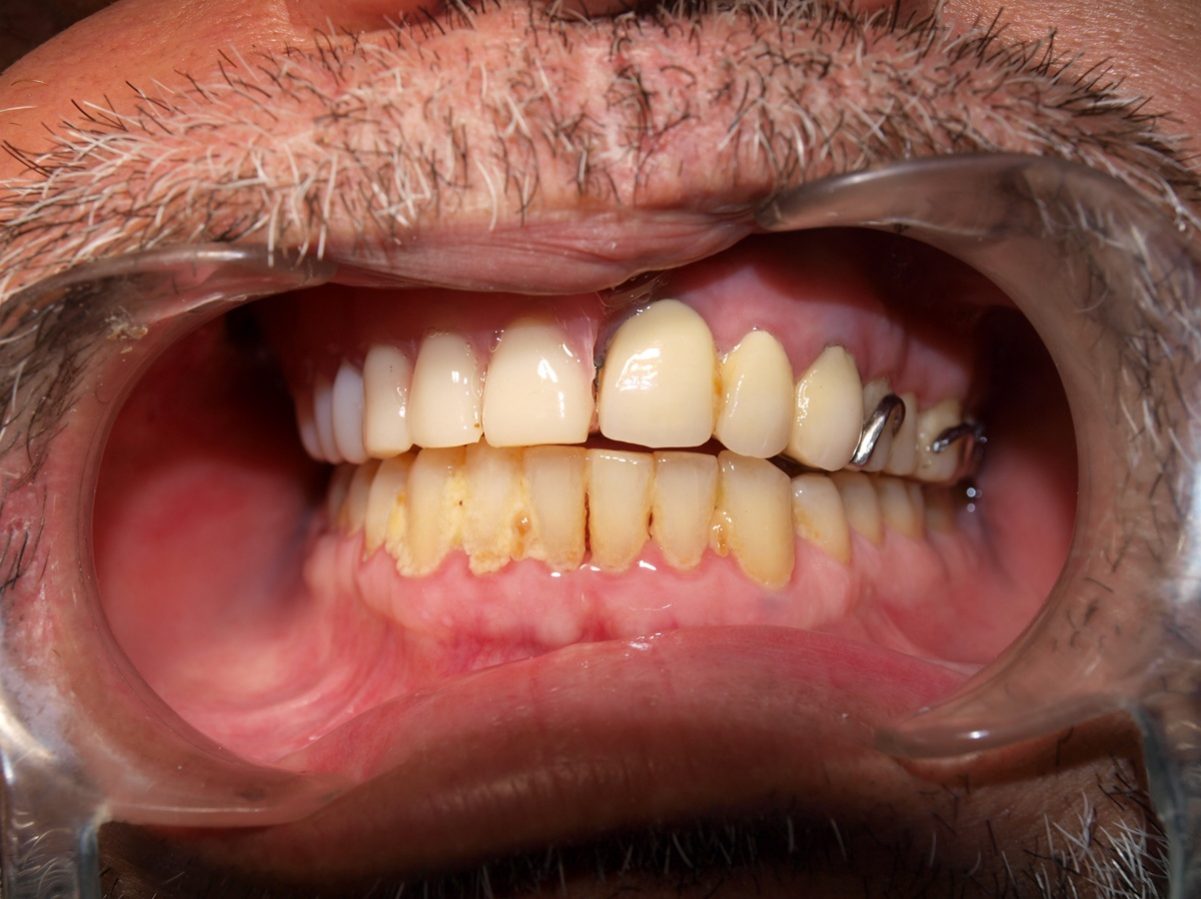
Insertion of Final Prosthesis During insertion, the superior bulb is inserted first. The splinted metal ceramic prosthesis with attachment is luted and then the oral part is inserted. The oral part contains the inferior hollow bulb, artificial teeth and the female part of the attachment.

The alignment was verified by asking the patient to bite in a centric occlusion, as shown in Figure 8. The oral part of the obturator with the antral extension lifted the superior bulb that supported the drooped eyeball. The patient was educated on how to wear and remove the prosthesis. Post-insertion instructions were emphasized. He was advised to clean the prosthesis after every meal with a toothbrush and mild soap solution. He was advised to use a betadine gargle. On the 24-hour follow-up, he complained of soreness along the buccal surface of the defect and the labial flange. The sore spots were identified, adjusted, and reassessed for the patient’s comfort. Recall visits were scheduled one week and one month after the prosthesis insertion. During the one-week review, the patient had difficulty in mastication and complained of a dull pain in the antral part. Pressure points on the antral part of the bulb were identified and adjusted. At the one-month review, the patient was relatively comfortable with chewing food. However, he was not able to chew efficiently on the right side compared to the left side. This is a limitation, and it was explained to the patient that occlusal contacts were minimized on the defect side intentionally to avoid undue pressure. Review appointments revealed that the patient was satisfied with the comfort, fit, and appearance.

## Discussion

Prosthesis delivery to a maxillectomy patient improves the quality of life by eliminating nasal leakage, unintelligible speech, swallowing difficulties, impaired mastication, unesthetic appearance, and psychological suffering [5]. Surgical and prosthetic rehabilitation, when combined, can offer functional and esthetic improvements to patients with extensive defects [3]. The success of the obturator prosthesis is affected by the maxillectomy procedure, which influences the remaining supporting structures of the oral cavity, the extent of the reconstructed defect, and its severity. The maxillectomy depends on the extent and type of the lesion. A Type I partial maxillectomy is a resection of a single wall, a Type II subtotal maxillectomy is a resection of ≤5 walls, a Type IIIA total maxillectomy is a resection of all six walls with preservation of the orbital contents, a Type IIIB is a resection of all six walls with orbital exenteration, and a Type IV orbito-maxillectomy is a resection of five walls with orbital exenteration and preservation of the hard palate [6]. The prosthesis design for any type of maxillectomy depends on the following considerations: a) closure of oral cavity, b) provision of a stable base for the restoration of function, c) restoration of mid-face symmetry, and d) support of the orbital structures [7].

Retaining the oral part of an obturator is important and is achieved by various methods. Zygomatic implants can provide adequate anchorage in patients with deficient bone [8], especially in the rehabilitation of maxillary defects, but this option was eliminated due to inadequate bone tissue in the zygoma. In clinical scenarios where implants cannot be used as retentive aids, there are other options for improved retention.

The attachment-retained cast partial denture with obturator was more esthetically appealing compared to a conventional cast partial design, which has a bar clasp on the anterior abutment teeth. In a two-piece hollow bulb obturator, the conventional design has a single antral hollow bulb that occupies the entire antrum of the defect and is self-retentive. The perforation on the antral bulb enables the patient to remove the bulb with ease. The inferior surface of the bulb fits into the indentation on the oral part that bears the teeth. The oral part of the obturator is made of cast partial denture framework with appropriate clasps, with or without precision attachments. In this case, a ball attachment on a splinted prosthesis over the existing teeth helped to retain the prosthesis along with the inferior hollow bulb. The resiliency of the ball attachment splinted to the rest of the remaining anterior teeth offers improved retention and preserves the remaining teeth by equally distributing the attachment forces [9]. Hollow obturators are a benefit to both the dentist and patient because of the reduced weight, hygiene facilitation, the ease of fabrication, and improved speech intelligibility. Wu and Scaaf showed that hollowing the obturator for partial maxillectomy patients significantly decreased the weight of the obturator by 6.55% to 33.06%, depending on the size of the defect [10]. In this clinical scenario, the fabrication of the framework design and the precision attachment is similar to a conventional two-piece hollow bulb obturator, but the technique for fabricating the antral bulb differs. The difference between the conventional bulb design and the split antral bulb is explained in Table 1.

**Table 1:** Differences Between the Conventional Bulb and the Split Antral Bulb

	Conventional two-piece Hollow Bulb	Split Hollow Bulb, two-piece
1.	Antral bulb occupies entire length of the antral portion.	Antral bulb occupies only the superior two-thirds of the antral portion.
2.	Oral part of the obturator has indentation on superior surface and makes passive contact with antral bulb.	Superior surface of oral part extends into inferior one-third of the antral portion.
3.	Does not support eyeball sagging.	Superior bulb provides acceptable support to eyeball.
4.	Oral part does not orient the antral bulb.	Antral bulb oriented in proper position while placing oral part of the obturator.
5.	Possibility of fluid leakage and food accumulation around indentation of oral part of the obturator.	Rare: Because of the extension of the oral part into part of the antral cavity, it approximates well with the superior bulb, minimizing the gap and hindering the accumulation of food.

**Figure 9:**
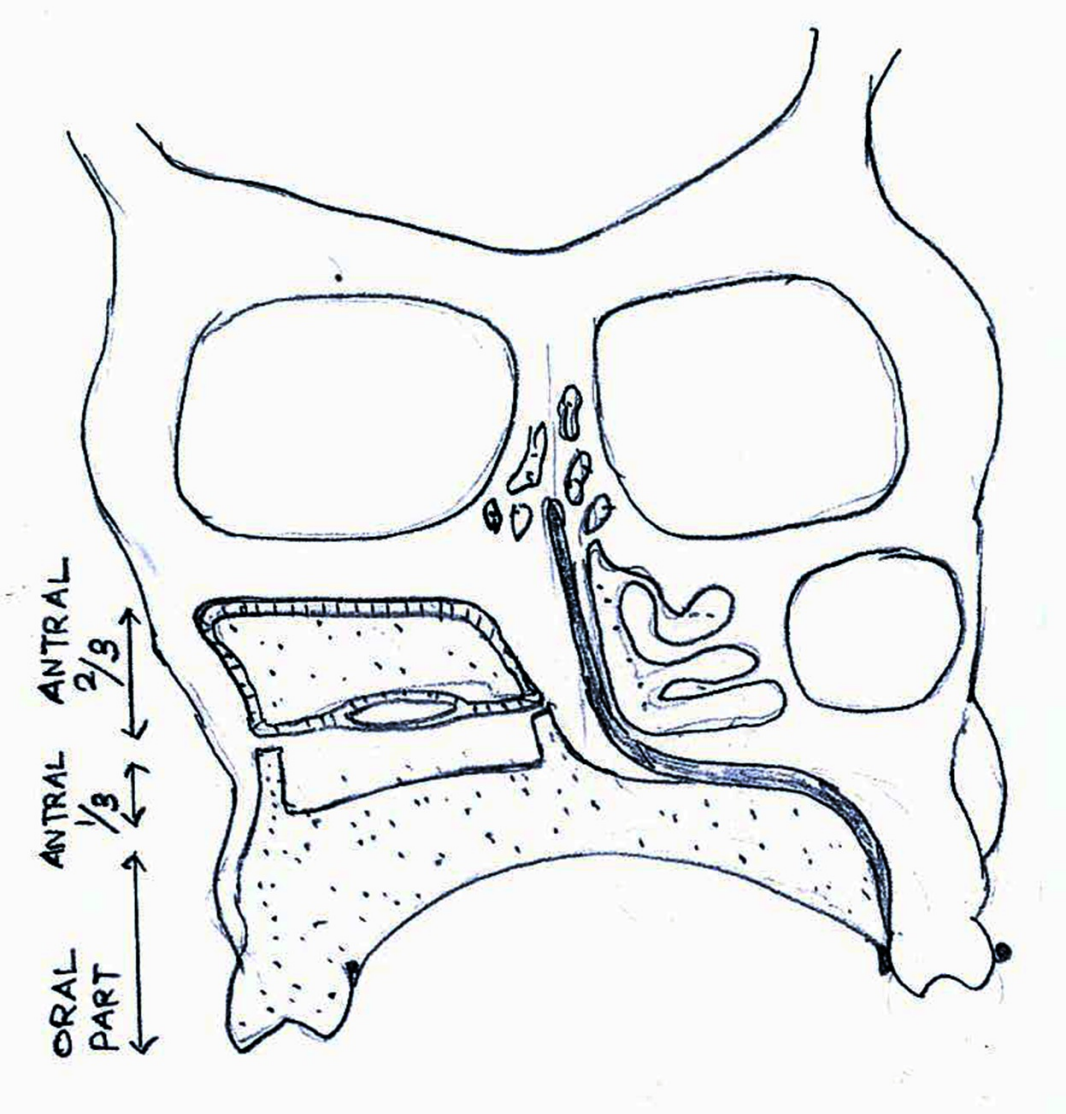
The Split Hollow Bulb Design This illustration portrays the new design adopted for this patient. It shows how the bulb design was modified to improve the retention and how it differs from conventional design.

The bulb design for this patient is hollow and split into two parts. The first part occupies the superior two-thirds of the defect and is self-retentive utilizing the undercuts. The second part occupies the inferior one-third of the defect and is an extension of the oral part as shown in Figure 9.

**Figure 10:**
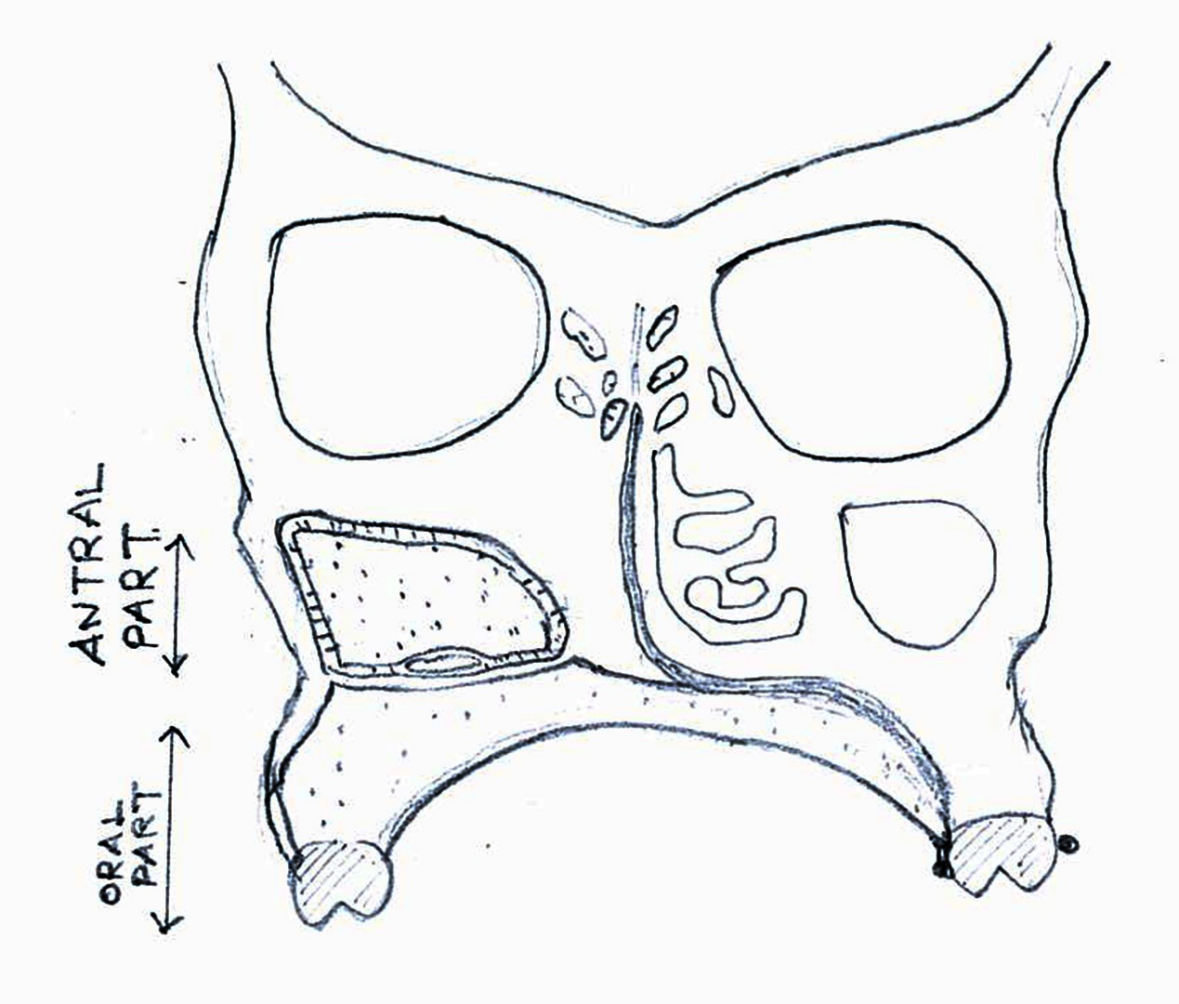
The Conventional Hollow Bulb Design

The primary significance of this split design is the inferior bulb, which extends from the oral part and stabilizes the superior antral bulb when the oral part or prosthesis is inserted. When positioned, the oral part lifts and repositions the superior bulb to provide eyeball support, minimizing drooping, whereas in a conventional design, the oral part will only have passive contact with the antral part and does not engage the defect area as shown in Figure 10.

This split design also offers facial support by minimally lifting the infraorbital region lost due to surgical excision. The close approximation also prevents fluid leakage and food accumulation around the defect area. However, this is technique-sensitive as the superior edges of the two bulbs should coincide with each other when inserted. Periodic recall appointments must be scheduled to evaluate the fit of the split bulb and oral prosthesis. A six-month review revealed that the prosthesis fit was satisfactory, and the patient had an acceptable level of oral hygiene. He was instructed to come in six months later (for a one-year review) for a relining of the antral extension of the obturator if needed.

## Conclusions

This custom-made obturator design met the patient’s needs for a greatly improved quality of life. More clinical reports involving such obturator designs, or periodic evaluations of masticatory efficiency (both subjective and objective) are required to validate this statement.
